# Inhibitors of cytoskeletal dynamics in malignant mesothelioma

**DOI:** 10.18632/oncotarget.27843

**Published:** 2020-12-15

**Authors:** Katarina Reis, Jack L. Arbiser, Anders Hjerpe, Katalin Dobra, Pontus Aspenström

**Affiliations:** ^1^Department of Microbiology, Tumor and Cell Biology, Karolinska Institutet, Stockholm, Sweden; ^2^Department of Dermatology, Emory University School of Medicine, Atlanta Veterans Administration Medical Center, Atlanta, GA, USA; ^3^Department of Laboratory Medicine, Division of Pathology, Karolinska Institutet, Huddinge, Stockholm, Sweden; ^4^Rudbeck Laboratory, Department of Immunology, Genetics and Pathology, Uppsala University, Uppsala, Sweden

**Keywords:** malignant mesothelioma, actin dynamics, vimentin, cytoskeleton, imipramine blue

## Abstract

Malignant mesotheliomas (MMs) are highly aggressive mesenchymal tumors that originate from mesothelial cells lining serosal cavities; i.e., the pleura, peritoneum, and pericardium. Classically, there is a well-established link between asbestos exposure, oxidative stress, release of reactive oxygen species, and chronic inflammatory mediators that leads to progression of MMs. MMs have an intermediate phenotype, with co-expression of mesenchymal and epithelial markers and dysregulated communication between the mesothelium and the microenvironment. We have previously shown that the organization and function of key cytoskeletal components can distinguish highly invasive cell lines from those more indolent. Here, we used these tools to study three different types of small-molecule inhibitors, where their common feature is their influence on production of reactive oxygen species. One of these, imipramine blue, was particularly effective in counteracting some key malignant properties of highly invasive MM cells. This opens a new possibility for targeted inhibition of MMs based on well-established molecular mechanisms.

## INTRODUCTION

Living organisms are formed by a number of cell types that work together to maintain normal tissue homeostasis. This neatly organized collaboration is lost in cancers, primarily because several cell-types responsible for correct tissue homeostasis change their behavior [1]. These changes are associated with alterations in cell morphology and cell migration, which are regulated by the cytoskeleton [2]. Malignant mesotheliomas (MMs) are rare but highly aggressive cancers, and they primarily originate from the pleura, peritoneum and pericardium [3]. There is a well-established link between asbestos exposure, release of reactive oxygen species (ROS) and inflammatory mediators, which collectively lead to malignant transformation of mesothelial cells and progression of MMs. However, there is also a significant number of cases with unknown etiology, in particular with peritoneal MMs [4].

Histological analysis has distinguished three phenotypic variants of MMs: epithelioid, sarcomatoid, and biphasic. Although all three of these are highly malignant, the epithelioid subtype has indications of better prognosis, whereas the sarcomatoid subtype is more fibroblast-like, which is considered a hallmark of poor prognosis [5, 6]. Direct invasion of the surrounding tissues is the main feature of MMs, which is caused by a dysfunctional communication between the mesothelium and the microenvironment. This communication is dependent on the dynamic organization of the cytoskeleton [2].

We have recently developed new tools for *in-vitro* analysis of cytoskeletal dynamics that correlate to tumor cell differentiation, which remains to date the only reliable predictive marker of malignant MM aggressiveness. These cytoskeletal features and dynamic rearrangements can potentially be used to monitor MM behavior. The three cytoskeletal filament systems are actin filaments, intermediate filaments and microtubules, and these are all critical for the control of cell morphogenesis, contraction, cell migration, and intracellular transport of vesicles and organelles [7]. The actin filament system is of particular importance for regulation of cell migration in health and disease [8]. In cancers, the organization and function of key cytoskeletal components are altered, and we have shown that careful analysis of these changes can provide clues to the malignancy grade of MMs. Early diagnosis of MMs and new diagnostic tools are urgently needed to effectively treat patients with MMs.

Asbestos is known to trigger inflammation and generation of ROS, which, in turn, can induce hypermethylation of tumor-suppressor genes. MMs tend to have high levels of NADPH oxidases, which generate superoxide, and which, in turn, inactivate *p53*, PTEN and IκB, thus leading to AKT and NFκB activation [9]. To this end, we investigated whether three types of compounds can counteract the malignant behavior of MM cells: imipramine blue (IB), honokiol (HKL), and Tris-dibenzylideneacetone-dipalladium (TDBA). The common denominator of these compounds is their known targeting of various aspects of ROS turnover [10–12]. STAV-FCS, STAV-AB, and ZL34 cells were all derived from pleural effusions of patients with MMs. Based on their overall morphology, STAV-AB cells have been classified as epithelioid, and STAV-FCS and ZL34 cells have been classified as biphasic [13]. Molecular profiling of these STAV-cell sublines has revealed profound molecular differences and dependencies on redox regulation [14–16].

We have previously shown that ZL34 cells are highly migratory and invasive in two-dimensional (2D) and 3D migration assays, whereas STAV-FCS cells show low migratory and invasive behaviors [Keller et al., submitted]. STAV-AB cells have a high 2D migratory potential, but they have a capacity for invasive growth between those of the ZL34 and STAV-FCS cells. For these reasons, we focused our study here on these three cell lines.

## RESULTS

### Effects of inhibitors on actin organization and cell morphology

**Figure 1:**
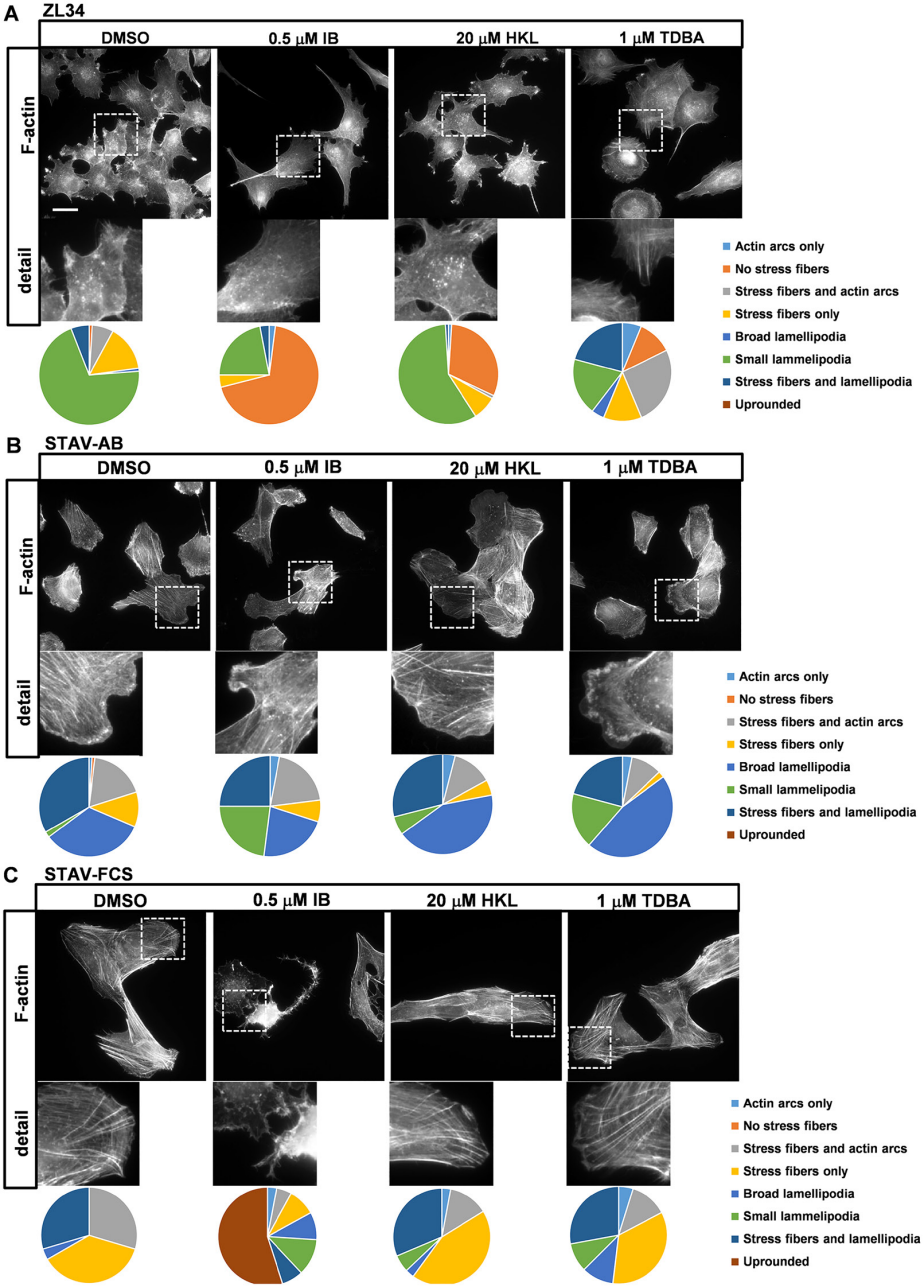
Effects of the inhibitors on organization of actin filaments. Representative images for filamentous actin (F-actin) visualized with TRITC-conjugated phalloidin in ZL34 (**A**), STAV-AB (**B**) and STAV-FCS (**C**) cells treated with 0.2% DMSO (vehicle control), 0.5 μM imipramine blue (IB), 20 μM honokiol (HKL) or 1 μM Tris-dibenzylideneacetone-dipalladium (TDBA) for 20 h. Scale bar, 20 μm. Analysis for actin organization, with pie charts showing relative effects on each aspect, as indicated. One hundred cells were examined per cell line, according to the dominant phenotype for each cell type, with the experiments performed three times.

Previously, we carried out systematic analysis of the differences in cytoskeletal organization and dynamics in eight MM cell lines representing different histological subtypes along the epithelial-to-sarcomatoid axis [Keller et al., submitted]. For the present study, we focused on three cell lines, which differed in their malignant properties. We tested three small-molecule inhibitors, IB, HKL, and TDBA, each of which has been shown to reduce cellular effects mediated by ROS production [10–12]. After testing a range of concentrations and time points (data not shown), the following working conditions were used for these MM cell lines: 0.5 μM IB, 20 μM HKL, and 1 μM TDBA. After 20 h of these treatments, the cells were fixed and their F-actin organization and cell morphology were analyzed (Figure 1 and Supplementary Figure 1).

ZL34 cells treated with DMSO only (as vehicle) had an abundance of small condensed lamellipodia (70.3% of cells) and stress fibers were common (27.7% of cells); in addition, actin dots were seen in the centers of the cells (Figure 1A). Treatment with 0.5 μM IB resulted in a loss of stress fibers (69.0% of cells) and actin dots, and decreased formation of small lamellipodia (22.0% of cells). Treatment with 20 μM HKL had no major impact on formation of small lamellipodia, but it resulted in stress fiber loss (31.1% of cells). Cells treated with 1 μM TDBA differed markedly from the control cells, as they showed more epithelial-like properties, such as broad lamellipodia and actin arcs, and they were more circular in appearance (Figure 1A and Supplementary Figure 1C).

The inhibitors had less prominent effects on the cell morphology and the actin organization in STAV-AB cells (Figure 1B and Supplementary Figure 1E–1H). Treatment with 0.5 μM IB resulted in a small shift from the formation of broad lamellipodia to small lamellipodia in STAV-AB cells, while treatments with 20 μM HKL and 1 μM TDBA only marginally affected their actin organization (Figure 1B).

In contrast to STAV-AB cells, treatment with 0.5 μM IB had substantial effects on STAV-FCS cell adhesion, as this resulted in widespread rounding up of the cells (55.0% of cells) and loss of stress fibers. Here, stress fiber content in the STAV-FCS cells decreased from the DMSO control treatment (93.5% of cells) to the IB treatment (21.0% of cells). However, treatments with 20 μM HKL or 1 μM TDBA had only marginal effects on the actin dynamics in these cells (Figure 1C).

### Effects of inhibitors on adhesion complex size

**Figure 2:**
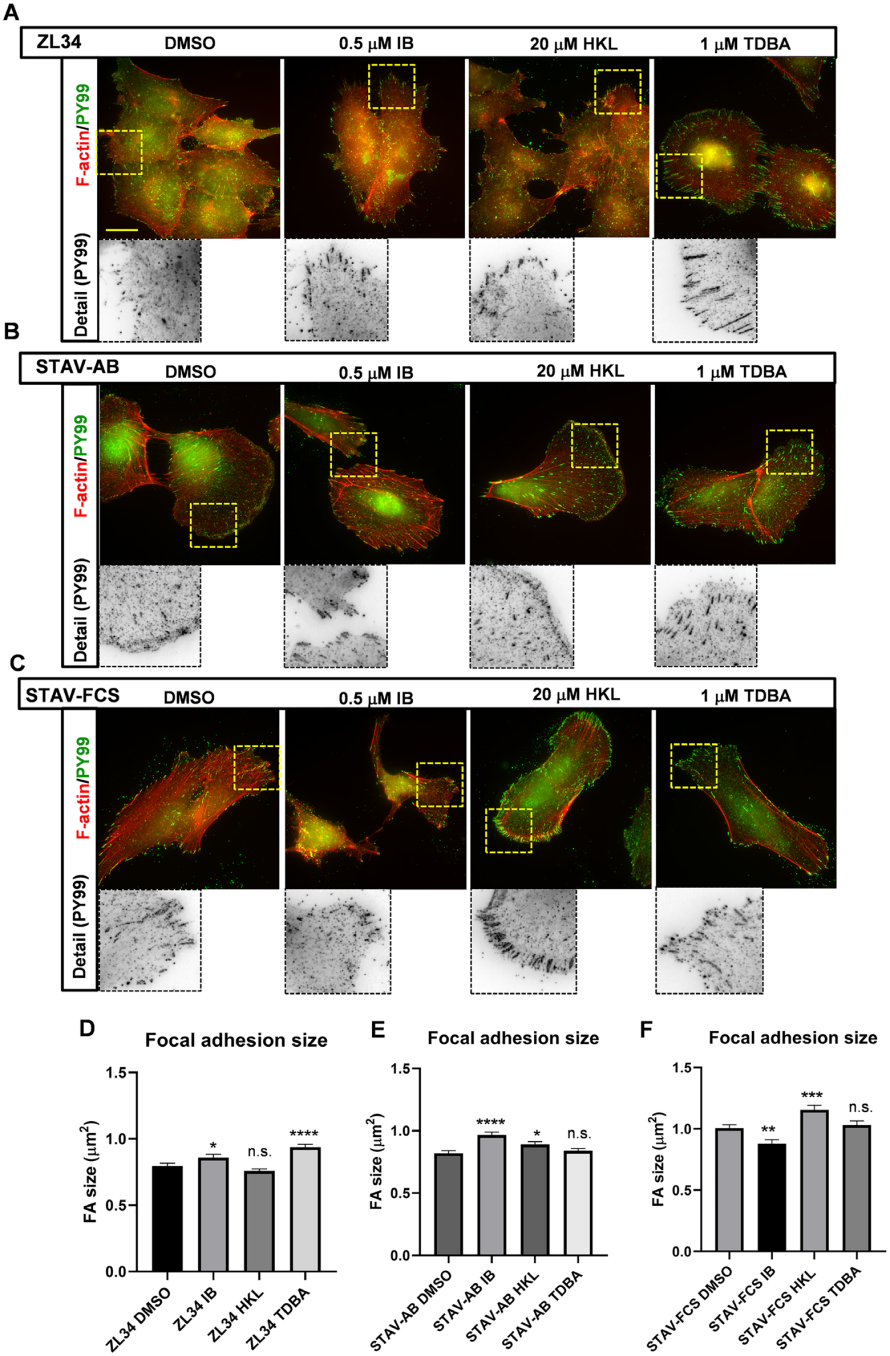
Effects of the inhibitors on organization of focal adhesions and focal contacts. (**A**–**C**) Representative images for filamentous actin (F-actin) visualized with TRITC-conjugated phalloidin, and for adhesion complexes stained with the mouse anti-phosphotyrosine (PY99) antibody followed by an AlexaFluor488-conjugated anti-mouse antibody. ZL34 (A), STAV-AB (B) and STAV-FCS (C) cells were treated with 0.2% DMSO (vehicle control), 0.5 μM imipramine blue (IB), 20 μM honokiol (HKL) or 1 μM Tris-dibenzylideneacetone-dipalladium (TDBA) for 20 h. Scale bar, 20 μm. (**D**–**F**) Quantification of focal adhesion sizes for ZL34 (D), STAV-AB (E) and STAV-FCS (F) cells treated as in (A–C), as assessed from microscopy images using ImageJ. ZL34 cells: DMSO (*n* = 2720), IB (*n* = 1941), HKL (*n* = 3897), TDBA (*n* = 3563); STAV-AB cells: DMSO (*n* = 2707), IB (*n* = 1979), HKL (*n* = 2469), TDBA (*n* = 3070); STAV-FCS cells: DMSO (*n* = 2426), IB (*n* = 1097), HKL (*n* = 1690), TDBA (*n* = 1570). Data are means ± standard error of the means. ^*^ *p* < 0.05; ^**^ *p* < 0.01; ^***^ *p* < 0.001; ^****^ *p* < 0.0001 (Student’s *t*-tests), *versus* relevant DMSO control. n.s. not significant.

We next analyzed the distribution and size of the cell:substrate attachment points in these three cell lines after treatment with these inhibitors. To this end, the cells were stained with an antibody against phosphotyrosine (PY99), which produces distinct staining of the adhesive structures. These are predominantly the well-developed focal adhesions (FAs), although focal contacts can also be visualized. The staining revealed that these ΜM cell lines had FAs at the ends of stress fibers and at the cell periphery (Figure 2A–2C). The sizes of the adhesion complexes were measured from microscopy images using the ImageJ software.

Treatment of ZL34 cells with 20 μM HKL did not lead to significant changes in FA size, but, interestingly, the FAs in the cells treated with 1 μM TDBA developed larger and more elongated FAs (control 0.80 vs. 0.94 μm^2^), which were similar to FAs in epithelioid MMs (Figure 2A and 2D) [Keller et al., submitted]. Treatment with 0.5 μM IB also led to an increase in FA size (0.80 vs. 0.86 μm^2^).

The FAs in STAV-AB cells treated with 0.5 μM IB were significantly larger than those in the control cells (0.82 vs. 0.97 μm^2^), which was probably a reflection of the loss of broad lamellipodia in these cells (Figure 2B and 2E). Treatment with 20 μM HKL resulted in a small, but significant, increase in FA sizes (0.82 vs. 0.89 μm^2^), whereas 1 μM TDBA did not produce any visible alterations in FA size (Figure 2B and 2E).

The FAs in STAV-FCS cells treated with 0.5 μM IB decreased significantly in size (1.01 vs. 0.88 μm^2^; Figure 2C and 2F). This would appear to be a result of the decrease in stress fibers and decreased adhesion of these cells, as shown in Figure 1C. In contrast the 20 μM HKL treatment increased FA size in STAV-FCS cells (1.01 vs. 1.16 μm^2^), whereas the FAs were refractory to 1 μM TDBA treatment (Figure 2C and 2F).

### Effects of inhibitors on vimentin localization

The subcellular localization and organization of vimentin filaments were previously shown to be altered in cancers [17]. We therefore studied the impact on the organization of vimentin by these inhibitors.

**Figure 3:**
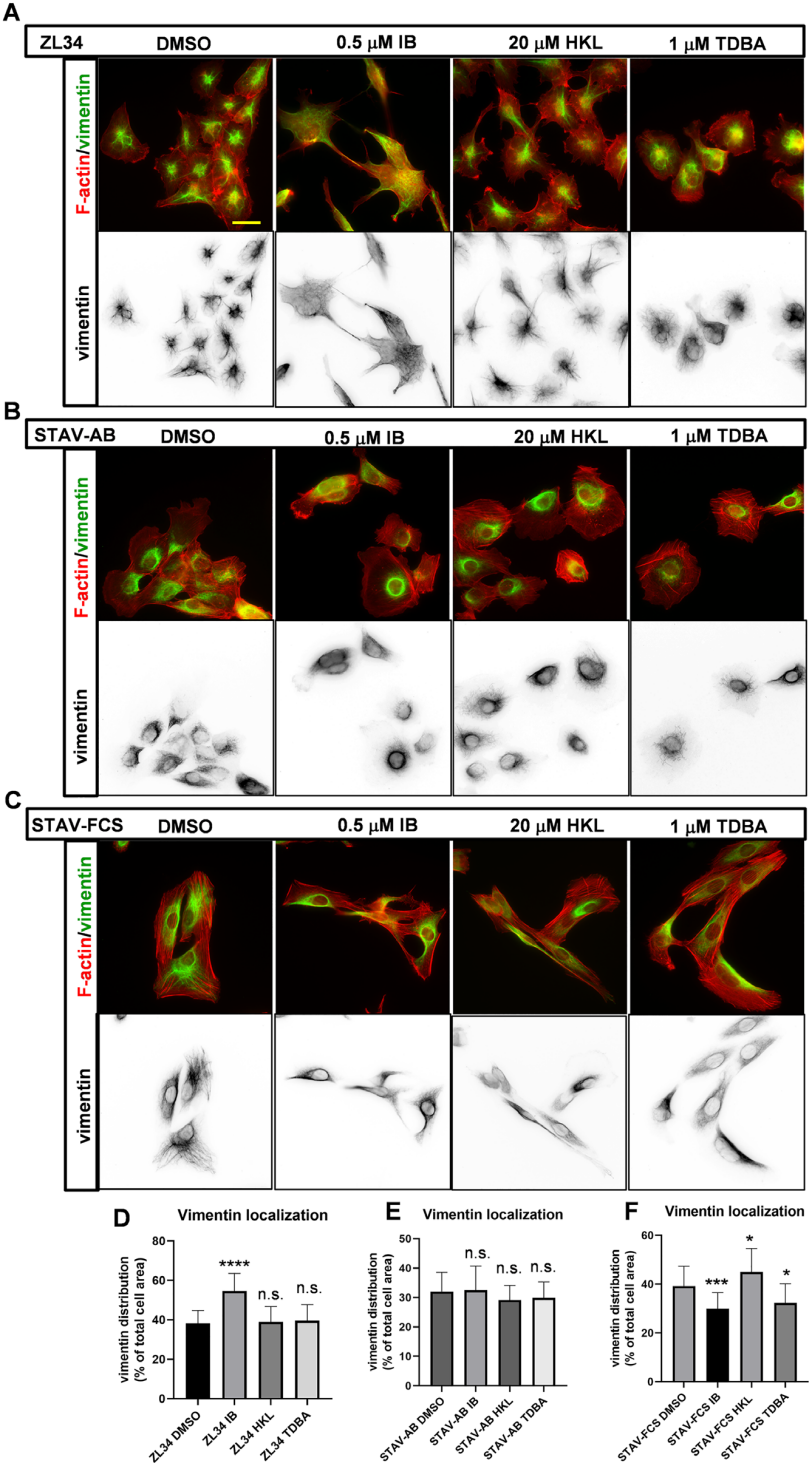
Effects of the inhibitors on the organization of vimentin filaments. (**A**–**C**) Representative images for vimentin intermediate filaments visualized with a mouse anti-vimentin antibody followed by an AlexaFluor488-conjugated anti-mouse antibody. Filamentous actin visualized with TRITC-conjugated phalloidin. ZL34 (A), STAV-AB (B) and STAV-FCS (C) cells were treated with 0.2% DMSO (vehicle control), 0.5 μM imipramine blue (IB), 20 μM honokiol (HKL) or 1 μM Tris-dibenzylideneacetone-dipalladium (TDBA) for 20 h. Scale bar, 20 μm. (**D**–**F**) Quantification of cell areas occupied by vimentin filaments for ZL34 (D), STAV-AB (E) and STAV-FCS (F) cells treated as in (A–C), as assessed by microscopy imaging. Ten images from three independent experiments for each cell line were analyzed using ImageJ. Data are means ± standard deviation. ^*^ *p* < 0.05; ^***^ *p* < 0.001; ^****^ *p* < 0.0001 (Student’s *t*-tests), *versus* relevant DMSO control. n.s. not significant.

The most obvious effects on vimentin organization was in ZL34 cells treated with 0.5 μM IB. In DMSO-treated cells the vimentin filaments appeared in an aster-like organization, and the filaments appeared to be in bundles that stretched out to the cell periphery (Figure 3A). The estimated area occupied by vimentin for this control was just over a third (38.3%) of the total cell area (Figure 3A and 3D). In contrast, cells treated with 0.5 μM IB showed very different organization of vimentin filaments: instead of the aster-like bundles, individual vimentin filaments appeared to fill most of the cytoplasm, and there was a significant increase in the area occupied by vimentin (54.6%) (Figure 3A and 3D). The treatments with 20 μM HKL and 1 μM TDBA did not significantly affect the vimentin organization (Figure 3A and 3D).

In STAV-AB cells, vimentin filaments were essentially organized in the perinuclear area, and they occupied a smaller area of the cells (32.0%) (Figure 3B and 3E) compared to ZL34 cells. None of the treatments with the three inhibitors visibly affected this vimentin organization.

In control STAV-FCS cells, vimentin filaments occupied a similar area (39.2%) (Figure 3C and 3F) to ZL34 cells. Here, treatment with 0.5 μM IB resulted in decreased area of vimentin (29.9%) (Figure 3C and 3F). For the 20 μM HKL and 1 μM TDBA treatments, there were small but significant effects on the vimentin organization (45.0%, 32.3%, respectively) (Figure 3C and 3F).

### Effects of inhibitors on YAP nuclear localization

Several lines of evidence link the Hippo pathway to dysfunction of the YAP transcription factor to cancers [18, 19]. We have previously seen that ΜM cells with higher migratory and invasive capacities have larger fractions of nuclear YAP [Keller et al., submitted]. To this end, we sought here to investigate the effects on YAP nuclear localization by these treatments with IB, HKL and TDBA.

**Figure 4:**
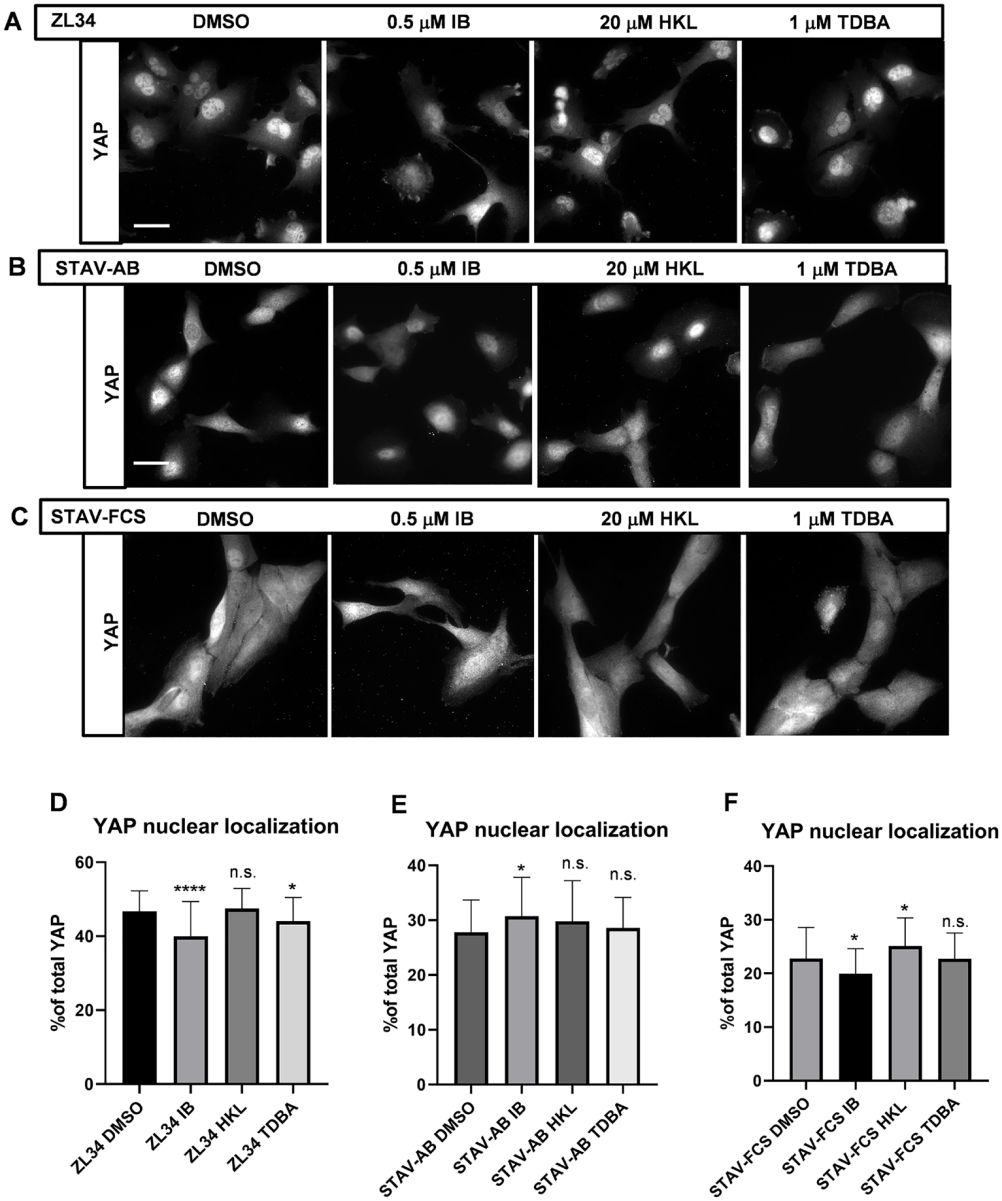
Effects of the inhibitors on the YAP nuclear localization. (**A**–**C**) Representative images for YAP nuclear localization analyzed by immunofluorescence microscopy after visualization with a mouse anti-YAP antibody followed by an AlexaFluor488-conjugated anti-mouse antibody. ZL34 (A), STAV-AB (B) and STAV-FCS (C) cells were treated with 0.2% DMSO (vehicle control), 0.5 μM imipramine blue (IB), 20 μM honokiol (HKL) or 1 μM Tris-dibenzylideneacetone-dipalladium (TDBA) for 20 h. Scale bar, 20 μm. (**D**–**F**) Quantification of proportions of nuclear YAP over the total cellular YAP in ZL34 (D), STAV-AB (E) or STAV-FCS (F) cells treated as in (A–C), as assessed from microscopy images using ImageJ. Data are means ± standard deviation. ^*^ *p* < 0.05; ^****^ *p* < 0.0001 (Student’s *t*-tests) *versus* relevant DMSO control. n.s. not significant.

The treatments had some variable effects on YAP nuclear localization. In particular, treatment of ZL34 cells with 0.5 μM IB resulted in a decrease in the fraction of nuclear YAP (46.7% vs. 40.0%) (Figure 4A and 4D). For the other inhibitors, the effects were milder, although with some significant changes seen: a small decrease for ZL34 cells with 1 μM TDBA (46.7% vs. 44.1%) (Figure 4A and 4D); a small increase for STAV-AB cells with 0.5 μM IB (27.8% vs. 30.7%) (Figure 4B and 4E); and variable for STAV-FCS cells, with a small decrease for 0.5 μM IB (22.8% vs. 20.0%) and a small increase for 20 μM HKL (22.8% vs. 25.1%) (Figure 4C and 4F).

### Effects on the microtubule organization and cell:cell contacts

We next analyzed the effects in these ΜM cell lines of the inhibitors in terms of the organization of microtubules and cell:cell contacts. Microtubule organization has been shown to differ in cancers [20].

However, there were no major effects in these cells of the inhibitors on microtubules, with the exception of one condition: ZL34 cells treated with 0.5 μM IB (Supplementary Figure 2). Generally, in all of the control cells with all of the treatments, the microtubules were more prominent in the area around the microtubule organizing center; in contrast, in these IB-treated ZL34 cells, the microtubules were more evenly spread through the cytoplasm (Supplementary Figure 2A).

Alterations in the function and localization of β-catenin have also been linked to cancers previously [21, 22]. Here, an antibody against β-catenin showed cell:cell contact areas in all three of these cell-lines (Supplementary Figure 3, DMSO). When they were treated with 0.5 μM IB, these cells all showed decreased cell:cell contact areas, with the most pronounced effect in ZL34 cells, where the cell:cell contacts all but disappeared (Supplementary Figure 3A). None of the cells under 20 μM HKL treatment showed any notable differences compared to their controls, although the cells exposed to 1 μM TDBA had markedly thinner cell:cell contact points (Supplementary Figure 3).

### Effects of the inhibitors on cell migration

Next, we investigated these MM cell lines for effects of these inhibitors on wound closure. In this case, an imaging system (IncuCyte) was used to produce wounds of the same size and shape, and to monitor the wound closure over extended periods of time (i.e., up to 5 days).

**Figure 5:**
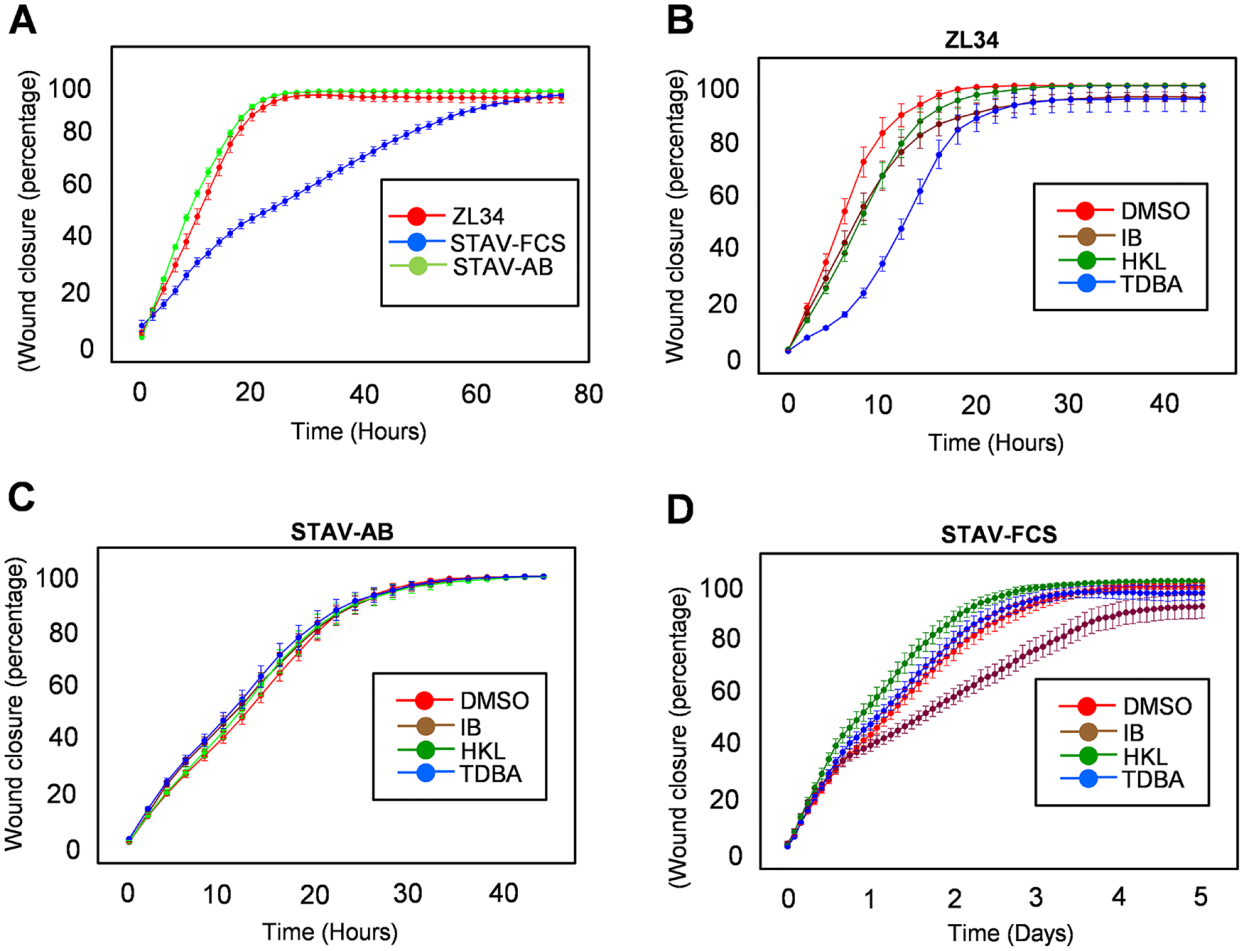
Effects of the inhibitors on the migratory properties. (**A**–**D**) Wound closure measures for migratory properties of ZL34 (A, B), STAV-AB (A, C) and STAV-FCS (A, D) cells for 80 h in the absence of treatments (A) and for 48 h in the presence (B–D) of 0.2% DMSO (vehicle control), 0.5 μM imipramine blue (IB), 20 μM honokiol (HKL) or 1 μM Tris-dibenzylideneacetone-dipalladium (TDBA), as assessed using an imaging device (IncuCyte).

Compared to ZL34 and STAV-AB cells, STAV-FCS cells showed slower wound closure (Figure 5A). The wound closure of ZL34 cells was decreased by all three inhibitors, where the effects of 0.5 μM IB and 20 μM HKL were less pronounced to the effect of 1 μM TDBA, which thus showed the greatest slowing of wound closure (Figure 5B). For the STAV-AB cells, the treatments with the inhibitors showed little or no slowing of wound closure (Figure 5C). Finally, the STAV-FCS cells showed marked slowing of wound closure with 0.5 μM IB, but were less affected by 20 μM HKL and 1 μM TDBA (Figure 5D).

## DISCUSSION

The number of drugs that can be used in the clinic for treatment of patients with MMs is currently very limited. The first-line chemotherapy approved by the US Food and Drug Administration is cisplatin together with an antifolate, such as pemetrexed or ralitrexed [23]. Similar effects can be obtained by combining carboplatin, liposomized doxorubicin and gemcitabine [24]. Identification of new treatments is very much needed, as each patient has an individual set-up of molecular alterations that are potentially targetable based on molecular phenotyping and by functional assays, such as those presented above [6]. A precision medicine approach is highly motivated for the most aggressive sarcomatoid phenotype, as the life expectancy for patients diagnosed with this subtype is particularly short. It is also of specific interest to search for potential novel drugs outside the common panel of chemotherapeutics, as surgery is contraindicated for the sarcomatoid subtype and the current chemotherapy regimens can only prolong patient life by about 3 months [23].

Oxidative stress is a known factor for development of MMs, particularly on the basis that iron in asbestos fibers has been suggested to catalyze the generation of free radicals [9]. This is one of the reasons why we sought to test compounds that can attack the production of ROS in MMs. To this end, three compounds were tested: IB, HKL, and TDBA. These compounds have been investigated for other cancers, but not for patients with MMs, with the exception of HKL [25].

Of these three agents, IB had the strongest effects on these MM cells. One reason for this appears to be because IB is an inhibitor of NADPH oxidase, and thereby it can have a direct negative impact on the production of ROS. IB has previously been used to impede glioma invasion [12]. In the present study, we show that IB had an impact on all MM cell lines tested, with its greatest effects on ZL34 cells. Treatment of these highly aggressive ZL34 cells with 0.5 μM IB for 20 h altered cell morphology, and reduced stress fiber content, lamellipodia formation, and 2D migration (i.e., delayed wound healing). IB treatment also resulted in increased FA size, altered vimentin filament organization, and reduced YAP nuclear localization, which are indicative of reduced malignant stage of these ZL34 cells. In contrast, for the STAV-AB cells, IB treatment did not have any significant effects. However, for STAV-FCS cells, IB also led to reduced stress fiber content, FA size, and 2D migration. This is in line with studies on glioma, where IB treatment resulted in loss of stress fibers [12]. Interestingly, similar differential effects in the more aggressive STAV-FCS cell line have been reported for selenite, a trace element with antioxidant and anticancer properties, where redox regulatory mechanisms were also involved [26].

Honokiol has been described as an activator of the mitochondrial histone deacetylase activator Sirtuin 3 (SIRT3) [11]. Treatment of ZL34 cells with 20 μM HKL for 20 h resulted in decreased actin bundles and reduced 2D migration, but had no effects on vimentin organization or YAP localization. STAV-AB and STAV-FCS cells were, however, not affected by this HKL treatment. Our data indicate that SIRT3 is not a key factor in the development of ΜMs. However, HKL has been shown previously to affect cell-cycle progression, which was seen as an increase in the sub-G1 population in a ΜM cell line. This cell-cycle effect was associated with suppressed expression of specificity protein 1 (Sp1) [25].

Tris-dibenzylideneacetone-dipalladium has been characterized as an inhibitor of N-myristoyltransferase-1 [10, 27]. This enzyme catalyzes the transfer of myristate to a number of target substrates, including the Src family kinases. Treatment of ZL34 cells with 1 μM TDBA for 20 h resulted in relocalization of actin filaments from stress fibers to actin arcs, and the cells appeared more rounded and epithelial-like. The sizes of their FAs increased, and appeared more similar to FAs associated with epithelioid MM cells. TDBA also decreased 2D migration of ZL34 cells. In contrast, the STAV-AB and STAV-FCS cells was more or less refractory to TDBA treatment, with the only significant effect seen for vimentin organization in STAV-FCS cells.

The data presented here show that IB, and to a lesser extent TDBA, can influence the malignant properties and behavior of MM cells. This was of particular significance for the highly malignant ZL34 MM cell line, but was also apparent for STAV-FCS cells. Collectively, our data indicate that these different MM cells are differentially responsive to compounds that have negative influences on oxidative stress. IB appears to be the most effective of these to counteract the malignant properties of the MM cells, although these cells at different stages of malignancies responded differentially to the treatments, where ZL34 cells were the most responsive. Inhibitors that act in the same way as IB have the potential to slow/ inhibit/ block cell transformation, which thus indicates their potential use for cancer therapies for patients with MMs. As well as the need for *ex-vivo* evaluation of cytotoxic drug responses in patient-derived MM cells [28, 29], such inhibitors of cytoskeletal dynamics will be tested further as a promising new class of agents that can modulate tumor aggressiveness, and can thus potentially improve survival of patients with MMs.

## MATERIALS AND METHODS

### Antibodies, reagents, and constructs

The following commercial antibodies and reagents were used: mouse monoclonal anti–α-tubulin (T9026); mouse monoclonal anti-vimentin (V6630); tetramethylrhodamine isothiocyanate (TRITC)-conjugated phalloidin (P1951) (Sigma-Aldrich); mouse monoclonal anti–β-catenin (#610153; BD Biosciences); mouse monoclonal anti-YAP (SC-101199) and mouse monoclonal anti-phosphotyrosine (PY99; SC-7020) (Santa Cruz); AlexaFluor 488-conjugated donkey anti-mouse (A21202) (Invitrogen-Molecular Probes). The following small-molecule compounds were used: IB, HKL, and TDBA, dissolved in dimethylsulfoxide (DMSO) [10–12].

### Cell culture, transfection, and immunofluorescence

For the human MM cell lines, the ZL34 and STAV-FCS cells were cultured in RPMI medium supplemented with 5% fetal bovine serum and 5% bovine serum, and the STAV-AB cells in RPMI medium supplemented with 10% human AB serum. All cultures were maintained at 37 °C in 5% CO_2_. For immunofluorescence analysis, the cells were fixed in 3% paraformaldehyde in phosphate-buffered saline (PBS) for 25 min at 37°C, and then washed in PBS. The cells were then permeabilized in 0.2% Triton X-100 in PBS for 5 min, washed in PBS, and incubated in PBS with 5% fetal bovine serum for 30 min at room temperature. The primary and secondary antibodies were diluted in PBS with 5% fetal bovine serum. The cells were incubated with the primary and secondary antibodies for 1 h for each, with washing in PBS. The coverslips were mounted on object slides using Fluoromount-G (Southern Biotechnology Associates). The cells were photographed under the microscope (AxioVert 40 CFL; Zeiss), which was equipped with a digital camera (AxioCAM MRm; Zeiss), and the AxioVision software was used.

### Single-cell migration with the scratch wound assay

Wound closure was monitored using the scratch wound assay (IncuCyte Zoom; Essen Bioscience). Here, the scratches were introduced using a wound maker, which creates wounds of equal width. Images were acquired under the microscopy every 2 h using a 10× phase-contrast objective. The scratch-wound analysis software (IncuCyte) allowed quantification of the increasing cell confluence inside the wound.

## SUPPLEMENTARY MATERIALS



## References

[1] Van Zijl F , Krupitza G , Mikulits W . Initial steps of metastasis: cell invasion and endothelial transmigration. Mutat Res. 2011; 728:23–34. 10.1016/j.mrrev.2011.05.002. 21605699 PMC4028085

[2] Sanz-Moreno V , Marshall CJ . The plasticity of cytoskeletal dynamics underlying neoplastic cell migration. Curr Opin Cell Biol. 2010; 22:690–696. 10.1016/j.ceb.2010.08.020. 20829016

[3] Jean D , Daubriac J , Le Pimpec-Barthes F , Galateau-Salle F , Jaurand MC . Molecular changes in mesothelioma with an impact on prognosis and treatment. Arch Pathol Lab Med. 2012; 136:277–293. 10.5858/arpa.2011-0215-RA. 22372904

[4] Sage AP , Martinez VD , Minatel BC , Pewarchuk ME , Marshall EA , MacAulay GM , Hubaux R , Pearson DD , Goodarzi AA , Dellaire G , Lam WL . Genomics and epigenetics of malignant mesothelioma. High Throughput. 2018; 7:pii:E20. 10.3390/ht7030020. 30060501 PMC6163664

[5] Tischoff I , Neid M , Neumann V , Tannapfel A . Pathohistological diagnosis and differential diagnosis. Recent Results Cancer Res. 2011; 189:57–78. 10.1007/978-3-642-10862-4_5. 21479896

[6] Hmeljak J , Sanchez-Vega F , Hoadley KA , Shih J , Stewart C , Heiman D , Tarpey P , Danilova L , Drill E , Gibb EA , Bowlby R , Kanchi R , Osmanbeyoglu HU , et al. Integrative Molecular Characterization of Malignant Pleural Mesothelioma. Cancer Discov. 2018; 8:1548–1565. 10.1158/2159-8290.CD-18-0804. 30322867 PMC6310008

[7] Huber F , Boire A , López MP , Koenderink GH . Cytoskeletal crosstalk: when three different personalities team up. Curr Opin Cell Biol. 2015; 232:39–47. 10.1016/j.ceb.2014.10.005. 25460780

[8] Davidson AJ , Wood W . Unravelling the actin cytoskeleton: a new competitive edge? Trends Cell Biol. 2016; 26:569–576. 10.1016/j.tcb.2016.04.001. 27133808 PMC4961066

[9] Benedetti S , Nuvoli B , Catalani S , Galati R . Reactive oxygen species a double-edged sword for mesothelioma. Oncotarget. 2015; 6:16848–16865. 10.18632/oncotarget.4253. 26078352 PMC4627278

[10] Bhandarka SS , Bromberg J , Carrillo C , Selvakumar P , Sharma RK , Perry BN , Govindarajan B , Fried L , Sohn A , Reddy K , Arbiser JL . Tris (dibenzylideneacetone) dipalladium, a N-myristoyltransferase-1 inhibitor, is effective against melanoma growth *in vitro* and *in vivo* . Clin Cancer Res. 2008; 14:5743–5748. 10.1158/1078-0432.CCR-08-0405. 18794083 PMC4423743

[11] Pillai VB , Samant S , Sundaresan N , Raghuraman H . Kim G , Bonner MY , Arbiser JL , Walker DI , Jones DP , Gius D , Gupta MP . Honokiol blocks and reverses cardiac hypertrophy in mice by activating mitochondrial Sirt3. Nat Commun. 2015; 6:6656. 10.1038/ncomms7656. 25871545 PMC4441304

[12] Munson JM , Fried L , Rowson SA , Bonner MY , Karumbaiah L , Diaz B , Courtneidge SA , Knaus UG , Brat DJ , Arbiser JL , Bellamkonda RV . Anti-invasive adjuvant therapy with imipramine blue enhances chemotherapeutic efficacy against glioma. Sci Transl Med. 2012; 4:127ra36. 10.1126/scitranslmed.3003016. 22461640

[13] Szulkin A , Nilsonne G , Mundt F , Wasik AM , Souri P , Hjerpe A , Dobra K . Variation in drug sensitivity of malignant mesothelioma cell lines with substantial effects of selenite and bortezomib, highlights need for individualized therapy. PLoS One. 2013; 8:e65903. 10.1371/journal.pone.0065903. 23840376 PMC3688685

[14] Sun X , Dobra K , Björnstedt M , Hjerpe A . Upregulation of 9 genes, including that for thioredoxin, during epithelial differentiation of mesothelioma cells. Differentiation. 2000; 66:181–188. 10.1046/j.1432-0436.2000.660404.x. 11269944

[15] Sun X , Wei L , Lidén J , Hui G , Dahlman-Wright K , Hjerpe A , Dobra K . Molecular characterization of tumour heterogeneity and malignant mesothelioma cell differentiation by gene profiling. J Pathol. 2005; 207:91–101. 10.1002/path.1810. 16007577

[16] Rundlöf AK , Fernandes AP , Selenius M , Babic M , Shariatgorji M , Nilsonne G , Ilag LL , Dobra K , Björnstedt M . Quantification of alternative mRNA species and identification of thioredoxin reductase 1 isoforms in human tumor cells. Differentiation. 2007; 75:123–132. 10.1111/j.1432-0436.2006.00121.x. 17316382

[17] Chou YH , Flitney FW , Chang L , Mendez M , Grin B , Goldman RD . The motility and dynamic properties of intermediate filaments and their constituent proteins. Exp Cell Res. 2007; 313:2236–2243. 10.1016/j.yexcr.2007.04.008. 17498691

[18] Gaspar P , Tapon N . Sensing the local environment: actin architecture and Hippo signaling. Curr Opin Cell Biol. 2014; 31:74–83. 10.1016/j.ceb.2014.09.003. 25259681

[19] Yeung B , Yu J , Yang X . Roles of the Hippo pathway in lung development and tumorigenesis. Int J Cancer. 2016; 138:533–539. 10.1002/ijc.29457. 25644176

[20] Cirillo L , Gotta M , Meraldi P . The elephant in the room: the role of microtubules in cancer. Adv Exp Med Biol. 2017; 1002:93–124. 10.1007/978-3-319-57127-0_5. 28600784

[21] Jamieson C , Sharma M , Henderson BR . Targeting the β-catenin nuclear transport pathway in cancer. Semin Cancer Biol. 2014; 27:20–29. 10.1016/j.semcancer.2014.04.012. 24820952

[22] Morgan RG , Ridsdale J , Tonks A , Darley RL . Factors affecting the nuclear localization of β-catenin in normal and malignant tissue. J Cell Biochem. 2014; 115:1351–1361. 10.1002/jcb.24803. 24610469

[23] Kelly RJ , Sharon E , Hassan R . Chemotherapy and targeted therapies for unresectable malignant mesothelioma. Lung Cancer. 2011; 73:256–263. 10.1016/j.lungcan.2011.04.014. 21620512 PMC3148297

[24] Hillerdal G , Sorensen JB , Sundström S , Riska H , Vikström A , Hjerpe AJ . Treatment of malignant pleural mesothelioma with carboplatin, liposomized doxorubicin, and gemcitabine: a phase II study. J Thorac Oncol. 2008; 3:1325–1331. 10.1097/JTO.0b013e31818b174d. 18978569

[25] Chae JI , Jeon YJ , Shim JH . Downregulation of Sp1 is involved in honokiol-induced cell cycle arrest and apoptosis in human malignant pleural mesothelioma cells. Oncol Rep. 2013; 29:2318–2324. 10.3892/or.2013.2353. 23525508

[26] Nilsonne G , Sun X , Nyström C , Rundlöf AK , Potamitou Fernandes A , Björnstedt M , Dobra K . Selenite induces apoptosis in sarcomatoid malignant mesothelioma cells through oxidative stress. Free Radic Biol Med. 2006; 41:874–885. 10.1016/j.freeradbiomed.2006.04.031. 16934670

[27] Díaz B , Ostapoff KT , Toombs JE , Lo J , Bonner MY , Curatolo A , Adsay V , Brekken RA , Arbiser JL . Tris DBA palladium is highly effective against growth and metastasis of pancreatic cancer in an orthotopic model. Oncotarget. 2016; 7:51569–51580. 10.18632/oncotarget.10514. 27438140 PMC5239497

[28] Szulkin A , Otvös R , Hillerdal CO , Celep A , Yousef-Fadhel E , Skribek H , Hjerpe A , Székely L , Dobra K . Characterization and drug sensitivity profiling of primary malignant mesothelioma cells from pleural effusions. BMC Cancer. 2014; 14:709. 10.1186/1471-2407-14-709. 25253633 PMC4190467

[29] Hillerdal CO , Ötvös R , Szatmári T , Own SA , Hillerdal G , Dackland ÅL , Dobra K , Hjerpe A . *Ex vivo* evaluation of tumor cell specific drug responses in malignant pleural effusions . Oncotarget. 2017; 8:82885–82896. 10.18632/oncotarget.20889. 29137310 PMC5669936

