# Supplementary material for: Inhibitors of cytoskeletal dynamics in malignant mesothelioma
Source: Oncotarget. 2020 Dec 15;11(50):4637–47. doi: 10.18632/oncotarget.27843 (PMC7747860; doi:10.18632/oncotarget.27843)
Supplement: Supplementary file 1 [file oncotarget-11-4637-s001.pdf]

Inhibitors of cytoskeletal dynamics in malignant mesothelioma

SUPPLEMENTARY MATERIALS

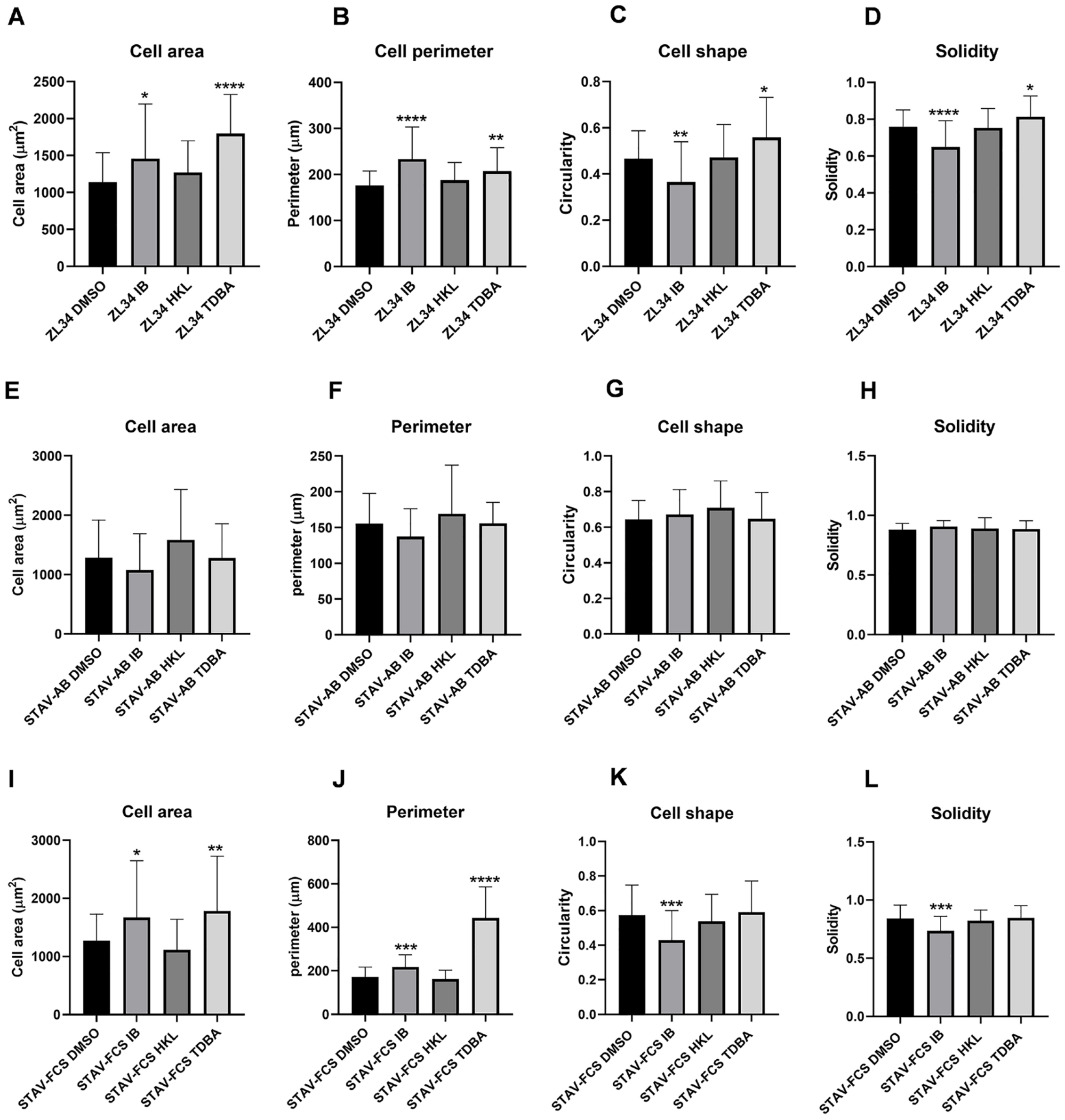

Supplementary Figure 1: Effects of the inhibitors on cell morphology. The cell size and morphology was examined by microscopy imaging of ZL34 (**A**–**D**), STAV-AB (**E**–**H**) or STAV-FCS (**I**–**L**) cells. The following shape factors were analyzed: cell shape (A, E and I), cell perimeter (B, F and J), circularity (C, G and K) and solidity (D, H and L). Ten images from three independent experiments for each cell line were analyzed using ImageJ. Data are means ± standard deviation. $^{*}p < 0.05$, $^{**}p < 0.01$, $^{***} = p < 0.001$ and $^{****}p < 0.0001$ (Student's *t*-tests) *versus* relevant DMSO control.

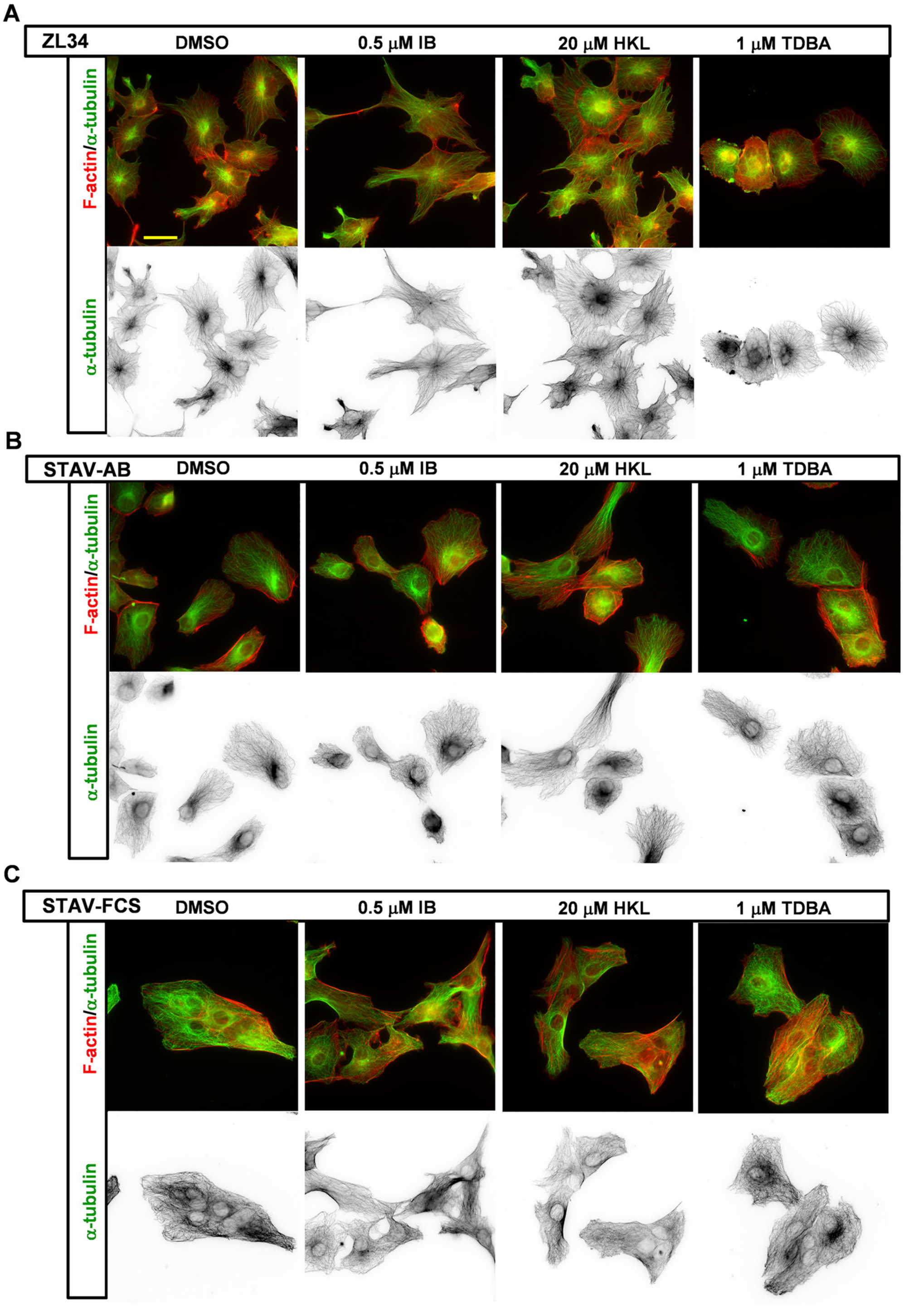

Supplementary Figure 2: Effects of the inhibitors on the organization of microtubules. Representative images for microtubule visualization with a mouse anti–α-tubulin antibody followed by an AlexaFluor488-conjugated anti-mouse antibody. Filamentous actin (F-actin) was visualized with TRITC-conjugated phalloidin. ZL34 (**A**), STAV-AB (**B**) and STAV-FCS (**C**) cells were treated with 0.2% DMSO (vehicle control), 0.5 μM imipramine blue (IB), 20 μM honokiol (HKL) or 1 μM Tris-dibenzylideneacetone-dipalladium (TDBA) for 20 h. Scale bar, 20 μm.

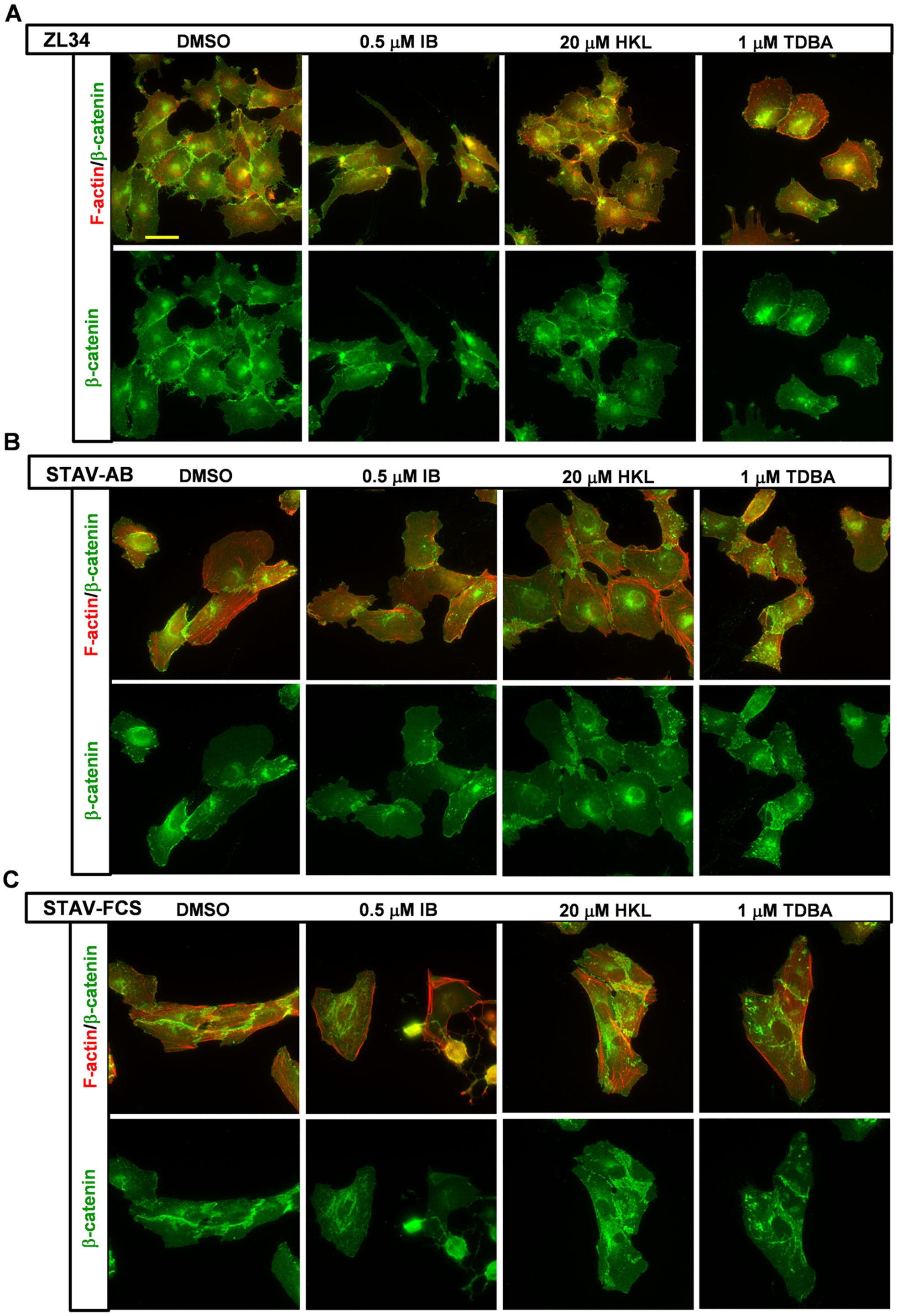

Supplementary Figure 3: Effects of the inhibitors on the organization of β-catenin. Representative images for cell:cell contact areas visualized with a mouse anti–β-catenin antibody followed by an AlexaFluor488-conjugated anti-mouse antibody. Filamentous actin (F-actin) was visualized with TRITC -conjugated phalloidin. ZL34 (**A**), STAV-AB (**B**) and STAV-FCS (**C**) cells were treated with 0.2% DMSO (vehicle control), 0.5 µM imipramine blue (IB), 20 µM honokiol (HKL) or 1 µM Tris-dibenzylideneacetone-dipalladium (TDBA) for 20 h. Scale bar, 20 µm.
